# The effect of human chorionic gonadotrophin contained in human menopausal gonadotropin on the clinical outcomes during progestin-primed ovarian stimulation

**DOI:** 10.18632/oncotarget.20508

**Published:** 2017-08-24

**Authors:** Xiuxian Zhu, Jing Ye, Yonglun Fu, Ai Ai, Renfei Cai, Yun Wang, Qingging Hong, Tian Hui, Qifeng Lyu, Qiuju Chen, Yanping Kuang

**Affiliations:** ^1^ Department of Assisted Reproduction, Shanghai Ninth People's Hospital, Shanghai Jiaotong University School of Medicine, Shanghai, P.R. China

**Keywords:** human menopausal gonadotropin, human chorionic gonadotropin, follicle stimulation hormone, progestin-primed ovarian stimulation, beta human chronic gonadtropin

## Abstract

Progestin-primed ovarian stimulation (PPOS) protocol has recently been demonstrated to be an novel regimen for preventing premature LH surges during controlled ovarian hyperstimulation (COH) in combination with frozen-thawed embryo transfer (FET). Our prospective controlled study was to explore the effect of human chorionic gonadotropin (hCG) contained in human menopausal gonadotropin (hMG) on the clinical outcomes in normalovulatory women undergoing COH with PPOS. A total of 180 patients were allocated into three groups according to the gonadotropin (Gn) used: group A (human menopausal gonadotropin, hMG-A), group B (hMG-B) or group C (follicle stimulating hormone, FSH). The primary outcome measured was the number of oocytes retrieved. The number of oocytes retrieved in group A B C was 10.72±5.78 11.33±5.19and13.38±8.97, respectively, with no statistic significance (p>0.05). Other embryological indicators were also similar (p>0.05). The concentration of serum and urinary β-hCG on the trigger day in group A and B were not associated with embryo results (p>0.05). There was no significant differences in the clinical pregnancy rate (41.67% vs. 51.56% vs. 39.51%, p>0.05) and implantation rate (31.58%vs. 34.75%vs.25.33%) after FET among the three groups. Thus the clinical characteristics were not affected by the hCG contained in hMG in normalovulatory women treated with PPOS.

## INTRODUCTION

Gonadotropin (Gn) has been used to induce the development of multiple follicles and increase the efficiency of ovarian stimulation in women with infertility since the 1960s. Originally, the human menopausal gonadotropin (hMG) with both follicle stimulating hormone (FSH) and luteinizing hormone (LH) activity was used. The overall LH activity in hMG was mainly derived from the human chorionic gonadotrophin (hCG), which was intentionally supplemented by manufacturers to standardize the product because of variable amounts of endogenous LH in hMG preparations during purification [1, 2]. The hCG plays a role similar to LH and contributes to folliculogenesis and oocyte maturation by modifying the steroid and protein micro- and macro-environments by binding to the LH/hCG receptor [3]. However, hCG has a higher number of glycosylated residues that result in a longer serum half-life (2.32 days for hCG vs 1 h for LH) and enhanced biological activity (hCG: LH activity of 6:1) [4–6]. Since some studies suggested that hCG components in hMG were detrimental to follicle growth and oocyte maturation, there was once a gradual shift from hMG to recombinant human FSH (r-FSH) [1, 7]. In contrast, some researcher observed that hCG alone was able to induce follicular growth instead of FSH [8], meanwhile, a beneficial effect of adding low-dose hCG to FSH was reported in patients with hypogonadotropic hypogonadism [9], polycystic ovarian syndrome [10] or poor ovarian response [11]. Thus the administration of exogenous LH-activity components was still controversial in the GnRH agonist or antagonist protocol.

Thanks to “freeze all” strategy, the constraints associated with ovarian stimulation in relation to the potential harmful effects of the hormonal environment on endometrial receptivity can be avoided. New strategies for improving the practice and results of IVF attempts were innovated [12]. The new stimulation protocol named progestin-primed ovarian stimulation (PPOS) where exogenous progesterone is used from the early follicular phase to block the LH surge during controlled ovarian hyperstimulation (COH), has recently been demonstrated to obtain similar embryonic characteristics and pregnancy outcomes compared to standard GnRH agonist (GnRH-agonist) short protocol following frozen-thawed embryo transfer (FET) [13–17]. The progesterone formulation is user friendly as it can be orally administered in comparision with the requirement for repeated injections of GnRH agonist and antagonists. Also it is economical than the usage of GnRH agonist and antagonists in the procedure of COH, though whether the overall cost of the “freeze-all” strategy increased is still obscure in consideration of the fees to freeze and thaw embryos.

PPOS was different from GnRH agonist or antagonist protocol with distinct mechanism and an elevated progesterone levels throughout the COH. Moverover, the impact of hCG in Gn preparations on the clinical outcomes during PPOS has not been reported. Therefore, we conducted a prospective controlled cohort study in normal ovulatory women undergoing *in vitro* fertilization (IVF) or intracytoplasmic sperm injection (ICSI) treatments by PPOS. The clinical results including the hormone profile, embryo results and pregnant outcomes were compared in terms of the types of Gn, levels of β-hCG and LH in serum and urine.

## RESULTS

### Patient characteristics

**Figure 1:**
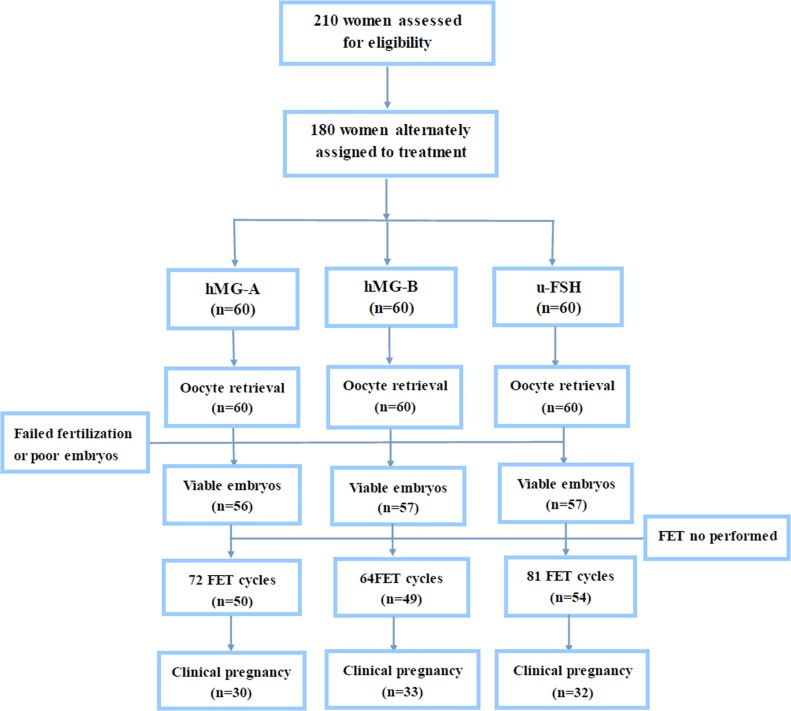
Study flow chart

Out of the 210 women that were assessed for eligibility, 180 were enrolled and assigned to the three experimental groups (Figure 1). In all 3 groups, the basic characteristics of the patients in the study including age, body mass index (BMI), the number of antral follicles, duration of infertility and basal endocrine characteristic were similar (Table 1).

**Table 1:** General information of patients

Index	hMG-A	hMG-B	u-FSH
Cycle (n)	60	60	60
Age (years)	31.53±3.38	31.63±3.57	31.3±3.92
Duration of infertility (y)	3.38±2.43	3.13±2.31	3.17±2.29
Body mass index (kg/m^2^)	20.38±4.51	20.47±4.44	21.14±3.84
Antral follicle count (n)	9.38±4.31	9.32±2.58	9.28±2.74
No. of previous transfer failures (n)	0.47±0.93	0.32±0.7	0.53±1.07
Base FSH [IU/L]	5.83±1.19	5.45±1.09	5.72±1.18
Base LH [IU/L]	3.25±1.2	3.37±1.55	3.84±1.79
Base E_2_[pg/ml]	32.92±15.08	33.51±13.84	31.2±19.28
Base progesterone [ng/ml]	0.3±0.13	0.3±0.17	0.25±0.1
Base β-hCG [ng/ml]	1.2	1.2	1.2

Values represent ($\bar{x}$ ± S).

### Ovarian stimulation, follicle development and oocyte performance

Table 2 shows the clinical and embryological characteristics of controlled ovarian hyperstimulation in the three groups. About 1-38 oocytes were retrieved and 1-23 embryos were cryopreserved among all 180 women. The mean duration of stimulation and gonadotropin doses were similar among the 3 groups (P>0.05). Group C had higher number of follicles (diameters larger than 10 mm) and number of retrieved oocytes than groups A and B (P>0.05). However, the rate of oocyte retrieval was higher in group B than in groups A and C (P<0.05). The numbers of D3 top-quality embryos and cryopreserved embryos, as well as cleavage rate and viable embryos per oocyte retrieved were similar among all three (P>0.05). Among the 180 women, 4 in the group A and 3 each in groups B and C had poor quality embryos or lacked fertilized oocytes. The cycle cancellation rate due to non-viable embryos was similar among the three groups (P>0.05).

**Table 2:** Stimulation and embryo characteristics of the patients

Index	hMG-A	hMG-B	u-FSH
No. of patients with ICSI	17	17	19
Serum FSH on the trigger day [IU/L]	16.16±4.79	17.46±3.93	14.55±5.05
Serum LH on the trigger day [IU/L]	1.52±1.22	1.54±1.33	1.39±1.08
Serum E2 on the trigger day [pg/ml]	2914.55±1366.52	3260.1±1282.54	2816.15±1586.22
Serum P on the trigger day [ng/ml]	0.59±0.28	0.68±0.35	0.66±0.5
Serum β-hCG on the trigger day [IU/L]	3.12±1.77^*^	1.9±0.73^*^	-
Uriany β-hCG on the trigger day [IU/L]	6.76±5.05^*^	4.43±3.01^*^	-
Total Gn dose (IU)	2055±347.2	2012.5±298.78	2066.25±327.77
Gn duration (days)	9.32±1.31	9.15±1.12	9.42±1.33
No. of >10mm follicles on the trigger day	12.67±5.68	12.45±5 25	15.4±9.66
No. of oocytes retrieved (n)	10.72±5.78	11.33±5.19	13.38±8.97
No. of MII oocytes (n)	9.4±4 99	10.23±4.89	12.28±8.45
No. of fertilized oocytes (n)	7.95±4.22	8.07±4.05	9.93±6.9
No. of cleaved embryos (n)	.78±4.14	7.92±4.0	9.75±6.71
No. of D_3_ top-quality embryos (n)	4.28±2.83	4.05±2.4	4.83±3.64
No. of viable embryos (n)	4.7±2.95	4.53±2.5	5.32±3 91
Oocyte retrieval rate (%)	65.95%# (643/975)	71.43% (680/952)	65.13%# (803/1233)
Cleavage rate (%)	97.9% 4 7/477)	98.14% (475/484)	98.15% (585/596)
Viable embryo rate per oocyte retrieved (%)	43.86% (282/643)	40% (272/680)	39.73% (319/803)
Cancellation rate (%)	6.67%(4/60)	5%(3/60)	5%(3/60)

^*^: P<0.05, group A/B compared with group C; values represent. (*X* ± S/%)

### Pregnancy outcomes

During the study, 180 women completed 217 FET cycles. Among these 47 finished 2, 7 finished 3 and one finished 4 FET cycles, respectively (Figure 1). The 401 embryos that were thawed were all viable. The endometrial preparation method, endometrial thickness on the day of transfer and the number of transferred embryos were comparable among the 3 groups. The clinical pregnancy rate among groups A, B and C was 41.67% (30/72), 51.56% (33/64) and 39.51% (32/81), respectively (P>0.05). There were 26 patients with twin pregnancies, which was highest in group A (40% vs. 24.24% vs. 18.75%). While 11 patients (2 in group A, 6 in group B and 3 in group C) experienced miscarriage before reaching the gestational age of 12 weeks, one patient had ectopic pregnancy. The implantation rate of embryos was comparable in the three groups, which indicated that the embryos had similar development potential (Table 3).

**Table 3:** Pregnancy outcomes during FET cycle

Index	hMG-A	hMG-B	u-FSH
No. of patients	50	49	54
No. of FET cycles	72	64	81
No. of thawed embryos	133	118	150
No. of viable embryos after thawed	133	118	150
No. of transferred embryos	1.83±0.38	1.83±0.38	1.85±0.36
**Endometrial preparation**			
Natural cycle	16	25	20
Mild stimulation	25	22	34
Hormone replacement therapy	31	17	27
Endometrial thickness (mm)	10.5±2.04	11.25±2.21	11.1±2.52
**Pregnancy outcome of FET**			
Biochemical pregnancy rate per transfer	47.22%(34/72)	57.81%(37/64)	48.15%(39/81)
Clinical pregnancy rate per transfer	41.67%(30/72)	51.56%(33/64)	39.51%(32/81)
Implantation rate	31.58%(42/133)	34.75%(41/118)	25.33%(38/150)
Miscarriage rate	6.67%(2/30)	18.18%(6/33)	9.38%(3/32)
Multiple pregnancy rate	40%(12/30)	24.24%(8/33)	18.75%(6/32)
Ectopic pregnancy rate	0%(0/30)	3.03%(1/33)	0%(0/32)
Intrauterine and ectopic pregnancy rate	0%(0/30)	3.03%(1/33)	0%(0/32)

### Hormone profiles

**Figure 2:**
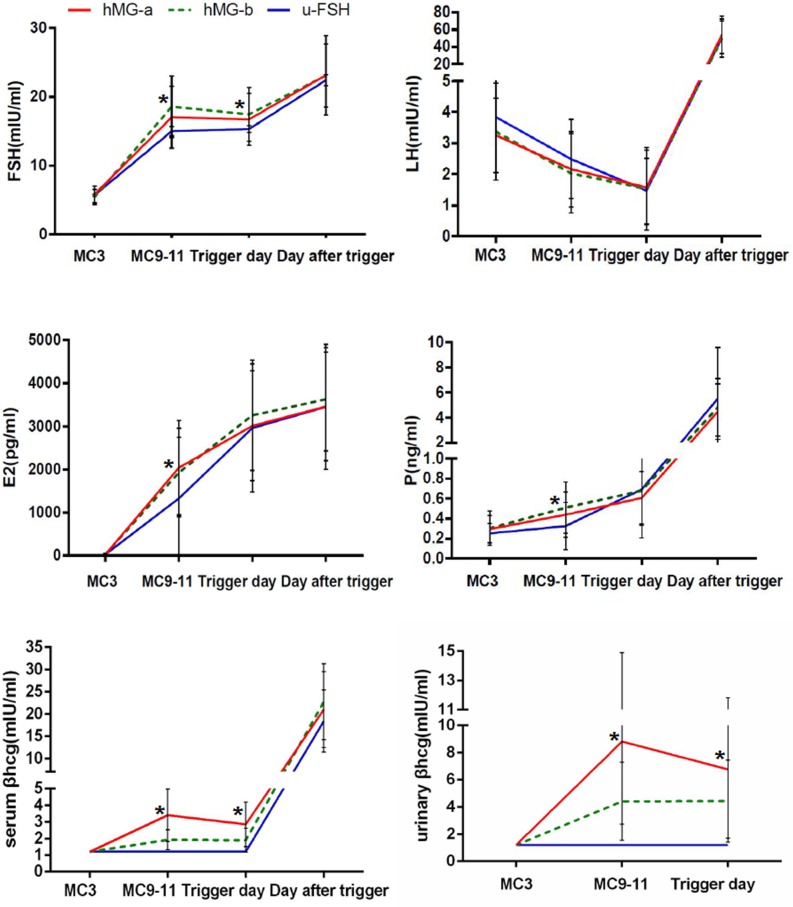
Serum hormone profiles during progestin-primed ovarian stimulation in hMG-A, hMG-B and u-FSH group patients The asterisk (^*^) denotes significant changes between groups at different time points (P<0.05).

The values of circulating concentrations of FSH, LH, E_2_, P and β-hCG in the three groups are presented in Figure 2. FSH levels were higher in hMG than in u-FSH groups on menstrual cycle day (MC_9-11_) and the trigger day (P<0.05). The LH values gradually decreased similarly in the three groups and no premature LH surges were detected during ovarian stimulation. The average LH levels were lower than the basal LH values in all the participants on the trigger day and increased on the day after trigger. The serum E_2_ concentrations on MC_9-11_ were higher in groups A and B (hMG) than group C (u-FSH), but were comparable on the trigger day and the next day. Progesterone values were low (0.31 ng/ml - 0.68 ng/ml) during ovarian stimulation, and increased after trigger with higher levels in group B than in groups A and C on MC_9-11_(P<0.05).

The serum and urinary β-hCG concentrations in group C were 1.2 IU/L (the lower limit of β-hCG measurement) throughout the ovarian stimulation period. The serum β-hCG concentrations group A were comparable on MC_9-11_ and the trigger day (3.49 ± 1.71 IU/L and 3.12 ± 1.77 IU/L) and were higher than in group B (1.93 ± 0.59 IU/L and 1.9 ± 0.73 IU/L; P<0.05). The urinary β-hCG concentrations in group A on MC_9-11_ and the trigger day were 8.81±6.09 IU/L and 6.76±5.05 IU/L, respectively, which was higher than in group B, which were 4.41±2.87 IU/L and 4.43±3.01 IU/L, respectively (P<0.05).

The serum and urine β-hCG concentrations correlated with one another (r=0.903, P<0.001) and were similar on MC_9-11_and the trigger day despite increased gonadotropin doses. In addition,there was no correlation between rate of mature oocytes and serum β-hCG (r=-0.152, P=0.099), urinary β-hCG (r=-0.061, P=0.510) and LH (r=-0.107, P =0.153) levels on trigger day among all participants.

### Subgroup analysis of embryo characteristics with LH or β-hCG levels

The women in groups A and B (hMG treated) were stratified into 3 groups namely, ≤P25, >P25–P75, and >P75 based on the 25th and 75th percentiles (P) of serum and urinary hCG levels on the trigger day. As shown in Tables 4 and 5, the three groups showed similar general characteristics and embryo features like number of cleaved embryos, the number of viable embryos, cleavage rate and viable embryo rate per oocyte retrieved (P>0.05). Similarly, when participants were divided into P25, >P25–P75, and >P75 groups based on serum LH levels on the trigger day, the general characteristics and embryo features like number of cleaved embryos, the number of viable embryos, cleavage rate and viable embryo rate per oocyte retrieved were comparable (P>0.05). However, as shown in Table 6, stimulation characteristics, the duration of stimulation, the total dose of gonadotropin, the number of oocytes retrieved, the number of MII oocytes and the number of fertilized oocytes were highest in the≤P25 quartile (P<0.05) and the number of oocytes retrieved were highest in the P25–P75 group (P<0.05).

**Table 4:** Subgroup analyses of menstrual cycle characteristics based on serum β-hCG values on trigger day

Index	<1.63 IU/L	1.63-3.16 IU/L	>3.16 IU/L
Cycle (n)	30	60	30
Age (years)	31.3±3.72	31.98±3.64	31.07±2.79
Duration of infertility (y)	2.97±1.79	3.47±2.6	3.13±2.4
Body mass index (kg/m2)	20.28±6.02	21.26±2.37	18.91±5.46
Antral follicle count (n)	10.17±3.82	9.15±3.07	8.93±4.09
No. of previous transfer failures (n)	0.3±0.6	0.3±0.72	0.67±1.12
hMG dose (IU)	1897.5±290.24	2050±354.12	2137.5±241.83
hMG duration (days)	8.8±1.06	9.3±1.29	9.53±1.11
No. of >10mm follicles on the trigger day (n)	12.23±5.23	12.18±5.02	13.63±6.44
No. of oocytes retrieved (n)	10.87±5.37	10.73±5.49	11.77±5.67
No. of MII oocytes (n)	9.83±5.11	9.67±4.83	10.1±5.11
No. of fertilized oocytes (n)	8.37±4	7.52±3.99	8.63±4.48
No. of cleaved embryos (n)	8.17±3.94	7.38±4.01	8.47±4.36
No. of viable embryos (n)	4.43±2.6	4.43±2.57	5.17±3.14
Cleavage rate (%)	98.06%(658/671)	98.19% (650/662)	94.64%(212/224)
Viable embryo rate per oocyte retrieved (%)	40.63%(364/896)	40.69% (376/924)	43.46%(133/306)

**Table 5:** Subgroup analysis of cycle characteristics based on urinary β-hCG levels on trigger day

Index	<2.76 IU/L	2.76-8.72 IU/L	>8.72 IU/L
Cycle (n)	30	60	30
Age (years)	33.3±3.75	31.08±3.3	30.72±2.94
Duration of infertility (y)	3.23±2.47	2.98±2.13	3.86±2.71
Body mass index (kg/m2)	21.65±2.56	20.02±5.25	19.91±4.13
Antral follicle count (n)	10.03±3.43	8.97±3.48	9.34±3.81
No. of previous transfer failures (n)	0.2±0.41	0.38±0.8	0.62±1.12
hMG dose (IU)	1937.5±329.63	2037.5±353.72	2125.86±221.65
hMG duration (days)	8.93±1.31	9.27±1.25	9.48±1.02
No. of >10mm follicles on the trigger day (n)	11.23±5.57	13.43±5.6	12.03±4.9
No. of oocytes retrieved (n)	10.07±6.15	11.7±5.45	10.62±4.85
No. of MII oocytes (n)	8.93±5.55	10.57±4.82	9.21±4.48
No. of fertilized oocytes (n)	6.87±4.2	8.67±4.09	7.86±4.01
No. of cleaved embryos (n)	6.7±4.19	8.5±4.09	7.72±3.87
No. of viable embryos (n)	4.07±2.8	4.85±2.8	4.62±2.48
Cleavage rate (%)	97.57%(201/206)	98.08% (510/520)	98.25% (224/228)
Viable embryo rate per oocyte Retrieved (%)	40.4% (122/302)	41.45% (291/702)	43.51% (134/308)

**Table 6:** Subgroup analysis of cycle characteristics based on LH values on the trigger day

Index	<0.68 IU/L	0.68-2.02 IU/L	>2.02IU/L
Cycle (n)	45	90	45
Age (years)	31.2±3.57	31.1±3.6	32.56±3.56
Duration of infertility (y)	3.02±2.34	3.01±2.31	3.87±2.31
Body mass index (kg/m^2^)	19.95±4.69	21.06±4.75	20.58±2.31
Antral follicle count (n)	9.58±2.86	9.62±3.33	8.71±3.75
No. of previous transfer failures (n)	0.49±0.99	0.43±0.88	0.4±0.92
Total Gn dose (IU)	2163.33±270.87^*^	2055±339.68	1905±293.71
Gn duration (days)	9.64±1.25^*^	9.4±1.22	8.73±1.18
No. of>10mm follicles on the trigger day (n)	15.53±7.08^*^	13.77±7.41	10.96±6.38
No. of oocytes retrieved (n)	13.33±6.33#	17.37±10.25	14.73±9.43#
No. of MII oocytes (n)	12.4±6.21^*^	10.48±6.28	9.2±6.59
No. of fertilized oocytes (n)	10.31±5^*^	8.39±5	7.51±5.76
No. of cleaved embryos (n)	10.09±4.85^*^	8.24±4.93	7.36±5.65
No. of viable embryos (n)	5.62±3.68	4.71±2.88	4.36±3.12
Cleavage rate (%)	97.84%(454/464)	98.28%(742/755)	97.93%(331/338)
Viable embryo rate per oocyte retrieved (%)	42.17%(253/600)	40% (424/1060)	42.06%(196/466)

^*^: P<0.05, group A/B compared with group C;

#: P<0.05,groups A & C compared with group B

Then, we compared the embryo characteristics in patients with LH<0.68 IU/L in groups A, B and C that were administered different concentrations of gonadotropin. As shown in Table 7, we observed that the number of antral follicles were higher in group B (10.75±1.84) than in groups A (8.2±3.34) and C (9.71±2.79). Meanwhile, the number of oocytes retrieved were highest in group C (P>0.05) and the viable embryo rate per oocyte retrieved was highest with group A (P<0.05). The other parameters were similar among the 3 groups.

**Table 7:** Subgroup analysis of cycle characteristics based on the type of Gn used in patients with LH<0.68 IU/L on the trigger day

Index	hMG-A	hMG-B	u-FSH
Cycle (n)	15	16	14
Age (years)	29.87±2.75	32.31±3.42	31.36±4.22
Duration of infertility (y)	3.6±2.82	3±1.97	2.43±2.17
Body mass index (kg/m^2^)	19.71±5.61	19.9±5.63	20.26±1.97
Antral follicle count (n)	8.2±3.34^*^	10.75±1.84	9.71±2.79
No. of previous transfer failures (n)	0.67±1.18	0.31±0.7	0.5±1.09
Serum β-hCG level on trigger day (IU/L)	3.98±2.26	2.19±0.68	—
Urinary β-hCG level on trigger day (IU/L)	8.92±6.14	3.87±2.07	—
Total Gn dose (IU)	2130±267.13	2235.94±301.52	2116.07±237.5
Gn duration (days)	9.47±1.19	9.94±1.34	9.5±1.23
No. of >10mm follicles on the trigger day (n)	12.33±4.55#	15±4.6	19.57±9.66
No. of oocytes retrieved (n)	11.2±4.6	12.69±3.77	16.36±8.99
No. of MII oocytes (n)	10.07±3.99	11.5±3.14	15.93±9.03
No. of fertilized oocytes (n)	8.53±3.4	10.25±2.84	12.29±7.41
No. of cleaved embryos (n)	8.33±3.4	10.19±2.88	11.86±7.12
No. of viable embryos (n)	5.07±3.13	5.37±2.28	6.5±5.29
Cleavage rate (%)	97.66%(125/128)	99.39%(163/164)	96.51%(166/172)
Viable embryo rate per oocyte retrieved (%)	45.24%#(76/168)	42.36%#(86/203)	39.74%(91/229)

^*^: P<0.05,group A /C compared with group B#: P<0.05, group A/B compared with group C

## DISCUSSION

This is the first prospective controlled cohort study investigating the effect of hCG in hMG on the clinical outcomes of normal ovulatory women undergoing IVF/ICSI treatments in the PPOS protocol. In general, the embryo quality and pregnancy outcomes were similar in patients treated with u-FSH as well as hMG. We also demonstrated it was more sensitive to detect individual differences in Gn metabolism by estimating β-hCG levels in urine than that in serum.

In our study, there were no significant differences in the pregnant outcomes after FET among the three groups. These results were similar to the meta-analysis that showed no substantive differences in efficacy of different gonadotropin sources [18]. However, our study results contradicted another study that showed higher clinical pregnancy rates upon hMG use [19]. A probable explanation for this contradiction could be the embryo transfer technique as the FET strategy may have decreased the beneficial impact of hCG on endometrium. This confirmed the meta-analysis that showed Gn types after FET did not influence clinical pregnancy rates [20]. In our study, some participants did not finish their FET cycles due to personal reasons that may have contributed to the bias in the data.

Our data showed no differences in the number of retrieved oocytes, mature oocytes and fertilization, cleavage and viable embryos among the three groups. A meta-analysis showed that hMG treatment resulted in fewer oocytes, higher total dose, and higher estrogen values than r-FSH treatment due to circulating levels of hCG derived from hMG injections on the human cumulus cells that hasten the demise of <14 mm follicles [21]. The mechanisms underlying these observations are not fully understood. However, major differences were observed in the gene expression profiles of pre-ovulatory granulosa cells after COH by FSH or hMG, thereby showing molecular evidence for mediation of the cumulus cells in embryo development [22].

There are no reports of the effect of LH levels on embryo characteristics in PPOS. Our study showed no premature LH surges. Thus, we compared the cycle characteristics by dividing patients into 3 different subgroups based on LH values on the trigger day. The data showed no differences in the number of cleaved embryos, the number of viable embryos, cleavage rate and viable embryo rate per oocyte retrieved (P>0.05) among the three subgroups. Interestingly, the number of MII oocytes and fertilized oocytes were highest in the ≤P25 quartile group (P<0.05), whereas the number of oocytes retrieved were highest in the P25–P75 group (P<0.05). One possibility is the differences in protocol. The LH suppression in the traditional down-regulation protocol and antagonist protocol were dose-dependent. However, the suppression of LH secretion in PPOS was a joint effect of progesterone, estrogen, and the GnRH neurosecretory system [23–24]. Thus, the over-inhibited LH level may be associated with higher E_2_ values produced by more oocytes. However, there was no association between the LH levels and the mature oocyte rate in our study. In addition, we need to be cautious due to the inclusion of patients treated with hMG, since its mechanism needs to be further investigated in a larger sample size.

In the subgroup of patients with LH values <0.68 IU/L on the trigger day, the number of oocytes retrieved was highest and the viable embryo rate per oocyte retrieved was least with u-FSH, as previously reported [25]. In contrast, low number of oocytes were retrieved from hMG-A patients although they also showed the highest viable embryo rate per oocyte retrieved with the average β-hCG level on trigger day being 3.98±2.26 IU/L in serum and 8.92±6.14 IU/L in urine. One explanation for these data could be the concept of ‘LH ceiling’, which suggests that LH values should neither be too high nor too low. This was in accordance with an hypothesis by Beretsos *et al* that hCG exposure throughout stimulation would facilitate embryo quality by mediating paracrine factors such as insulin-like growth factor-1 and transforming growth factor-β [26, 27]. Based on the principle of adequate LH levels, there may be an optimal situation of adding hCG. If a particular range of hCG in serum or urine correlated with increased rate of oocyte maturity, then hCG injections could be tailored to achieve those levels by determining serum and urine β-hCG levels. In our study, the embryo characteristics were similar in the three subgroups based on the type of gonadotropin used in patients with LH<0.68IU/L on the trigger day. This finding should be cautious due to the limited sample size.

The hCG levels vary among various commercial hCG assays (different combinations of 7 antibodies) because of multiple hCG-related metabolites in the serum and urine samples including degraded hCG, hyper and hypoglycosylated hCG, free α and β subunits and β-hCG core fragments [28]. In our study, the Abbott Architect system detected only β-hCG as the antibody combined with non-nicked hCG molecules and free β-hCG subunit without cross-reacting with the core β- fragment [28, 29]. The half-life of serum β-hCG was 3.93±0.68 h and was metabolized by the kidneys and accumulated in urine [28, 29], thereby adding to discrepancy in serum measurements. Thus, urinary β-hCG assayed by the Abbott Architect system reflected the absorptive capacity of hMG in different patients as previously described [30]. Our determination shows the urine β-hCG levels was always higher than the serum β-hCG levels, which indicates the sensitivity of urine in the detection of β-hCG. It can help distinguish the differences below 1.2 IU/L in serum and magnify the range of serum β-hCG levels. This may establish a new method to investigation in the usage of hCG.

Furthermore, there was no association between β-hCG levels and the rate of mature oocyte, probably due to varied β-hCG levels in patients. Elkind- Hirsch *et al.* showed different serum β-hCG levels despite similar doses of hCG; also, BMI was inversely correlated with β-hCG levels [31]. In contrast, we observed different hCG levels despite comparable BMI and similar hCG injections suggesting that hCG metabolism varied among individuals. Subgroup analysis based on different β-hCG levels in serum and urine suggested that higher β-hCG levels were associated with increased number of viable embryos. This was in accordance with a retrospective study by Arce *et al*, which demonstrated that mid-follicular concentrations of exogenous hCG originated from high purified hMG (HP-hMG) and positively associated with live-birth rate and the number of top-quality embryos in patients treated with GnRH agonist [32].

There are limitations related to the design of our study. First, the time between injection and measurement of hCG was variable in this study. A pharmacokinetics study demonstrated that serum β-hCG levels peaked at 12 h after injection and decreased over the course of 120 h thereafter [33]. Therefore, the optimal time to measure serum β-hCG levels was 12 h after injection. In our study, patients were usually injected in the evening (5-7pm) and blood was drawn in the morning (7-9am) on the next day and therefore was not exactly at 12 h. Second, the urine samples were collected without an exact collection time and restricted water intake, which could vary urinary β-hCG levels as reported earlier [34]. Third, the sample size was limited as u-FSH was not preferred in our clinic since its price was six times than hMG in China. Finally, incomplete FET cycles contributed to the reduced power of this study.

In conclusion, hMG treatment in normal ovulatory patients resulted in the same embryo quality and clinical pregnancy outcomes compared to FSH in the PPOS protocol irrespective of the discrepancies in serum and urine hCG values. Since the price of hMG is only one seventh of the price for FSH in China, our study permits clinical use of the cost-effective Gn preparation for patients undergoing COH. In addition, our study shows the urine is a more sensitive method to detect β-hCG levels than the serum which may be useful for researches related to hCG administration during COH. The sample size is limited as a preliminary trial, therefore, future prospective studies with larger patient pools are necessary to further optimize the PPOS regimen.

## MATERIALS AND METHODS

### Study setting and patients

We conducted this prospective controlled cohort study at the Department of Assisted Reproduction of the Ninth People's Hospital of Shanghai Jiaotong University School of Medicine by enrolling 180 women undergoing IVF/ICSI regimens for infertility treatment between September 2014 to December 2015. The study protocol was approved by the Ethics Committee (Institutional Review Board) of the Ninth People's Hospital of Shanghai and the trial was registered with the Chinese Clinical Trial Registry (ChiCTR-OPN-14005276). It was conducted according to the Declaration of Helsinki for medical research. All participants provided informed consent after counseling for infertility treatments and routine IVF procedures.

The inclusion criteria were (1) patient age was less than 40; (2) patients had a regular menstrual cycle over the previous three month period (25-35 days); (3) the antral follicle count was more than 5 on menstrual cycle days 2-3; and (4) the basal serum FSH concentration was no more than 10 IU/L.

Study exclusion criteria were (1) documented ovarian failure including basal FSH above 10 IU/L or antral follicles not found by ultrasound examination; (2) endometriosis grade 3 or higher; (3) diagnosed with polycystic ovarian syndrome (PCOS); (4) received hormonal treatments in the previous three months; (5) any contraindications to ovarian stimulation treatment; and (6) documented oocyte pick-up failure.

### Patient group allocation and sample size estimate

This was a prospective non-inferiority trial. Since the clinical characteristics of the PPOS protocol were unknown at the start of the study, it was not feasible to accurately estimate the sample size for this prospective study. It was decided to perform analysis when 150 patients had entered in the study. In consideration of potential dropouts, data analysis was performed when 180 patients had been randomized. Patients were randomly assigned in a 1:1 ratio to receive either types of gonadotropin by a computer generated drawing of random numbers assigned in a sealed envelope. The study researcher was blinded to the order of group assignment at the time of recruitment and randomization, whereas the physicians and embryologists involved in oocyte retrieval and embryo transfer were masked to group assignments of participants in the trial. Participants were not masked to group assignments.

## PROCEDURES

### Controlled ovarian stimulation

The 180 patients recruited for this study were allocated to three groups based on the type of Gn being used, namely (1) group A, hMG-A (brand name: fengyuan; Anhui Fengyuan Pharmaceutical Co., China); (2) group B, hMG-B (brand name: lebaode; Lizhu Pharmaceutical Trading Co., China); (3) group C, u-FSH (brand name: lishenbao, Lizhu Pharmaceutical Trading Co., China). The patients were administered 10 mg/day MPA and 225 IU Gn from MC_3_ onwards. Follicles were monitored from MC_9-11_ onwards every 2-4 days by a transvaginal ultrasound examination to record the number of developing follicles. When three dominant follicles reached 18 mm in diameter, the MPA and Gn treatment was stopped and the final stage of oocyte maturation was triggered by administering 0.1 mg triptorelin (Decapeptyl, Ferring pharmaceuticals, Germany) and 1000 IU hCG (Lizhu Pharmaceutical Trading Co., China). Transvaginal ultrasound-guided oocyte retrieval was performed 34-36 hrs after triggering oocyte maturation. All follicles ≥ 10 mm diameter were retrieved.

Fertilization was carried out by either IVF or ICSI depending on semen parameters. Embryos were examined on the third day and all good-quality embryos (including grade 1 and grade 2, 8-cell embryos) were frozen by vitrification. Other embryos were further cultured until they reached the blastocyst stage and blastocysts that showed normal morphology were frozen on days 5 or 6.

### Hormonal measurement

Serum FSH, LH, E_2_, progesterone, β-hCG levels were measured on MC_3_, MC _9-11_(after 6-8 days of stimulation), the day when oocyte maturation was triggered and the day after trigger (approximately 10 h after the injection of triptorelin, a GnRH agonist and hCG). Urinary β-hCG was measured on MC_9-11_ and the trigger day.

Hormonal levels were measured by chemiluminescence (Abbott Biologicals B.V, Netherlands). The Abbott Architect system used in our study detected only β-hCG, whose lower limit was 1.2 IU/L and sensitivity was 0.01 IU/L. The lower limits of sensitivity were as follows: FSH, 0.06 IU/L; LH, 0.09 IU/L; E_2_, 10pg/ml; progesterone, 0.1ng/ml and β-hCG, 1.2 IU/L. Since the upper limit of E_2_ measurement was 5000 pg/ml, the E_2_ values were recorded as 5000 pg/ml if the E_2_ levels on the trigger day or day after trigger were higher. Intra- and inter-assay coefficients of variation were 2.6% and 5.8% for FSH, 5.9% and 8.1% for LH, 6.3% and 6.4% for E_2_ and 7.9% and 10% for progesterone.

One ampoule hMG included 75IU FSH and 75IU LH activity. Data was not available for hCG activity in hMG since it was not a compulsory requirement of international pharmacopoeias. The activities in one ampoule of each of the three types of gonadotropin employed in our study were determined with Abbott Architect system. The β-hCG levels were 16.77 IU/L for hMG-A (fengyuan), 7.86 IU/L for hMG-B (lebaode) and 1.2 IU/L for u-FSH (lishenbao), respectively.

### Endometrium preparation and FET

Patients with thin endometrium, uterine polyps, submucous myoma (diameter no more than 10 mm) and previous IVF failures were advised to perform hysteroscopy screening prior to FET to detect and treat intra-uterine pathologies. Natural FET cycles were used for women with regular menstrual cycle and letrozole was administered for patients with irregular menstrual cycles. For patients with thin endometrium during natural or stimulation cycles, hormone replacement treatment with oral ethinyl estradiol (25μg tid or thrice a day) (Xinyi Pharmaceutical Co., China) was recommended from day 3 of the cycle for endometrial preparation. Once the endometrial lining reached thickness greater than 8 mm, estradiol and dydrogesterone (Abbott Healthcare Products B.V, Netherlands) and vaginal progesterone soft capsules (Laboratoires Besins International, France) were administered. Embryo transfer was performed three days later and blastocyst transfer was performed on the fifth day. Upon successful pregnancy, the progesterone supplement was continued until 10 weeks of gestation.

### Statistical analysis

The number of oocytes retrieved was the primary outcome measure whereas the number of mature oocytes, the rate of viable embryos, the clinical pregnancy rate after FET were secondary measures. Rate of viable embryos was defined as the number of viable embryos obtained divided by the number of oocytes retrieved. The implantation rate was defined as the number of gestational sacs divided by the number of embryos transferred. Clinical pregnancy was defined as the presence of a gestational sac with fetal heart activity during ultrasound examination at 7 weeks after FET. The miscarriage rate was defined as the proportion of patients with spontaneous termination of pregnancy.

Efficacy analyses of the primary and secondary end points was determined in treated subjects including those that did not obtain viable embryos after treatment cycles and did not have a clinical pregnancy. In the tables presented in this study, data are presented as the mean ± SD (x¯±S) and percentage (%). The normality of continuous variables was tested by the Shapiro-Wilk test. Continuous variables were compared via one-way ANOVA if the normality assumption was true; otherwise, the Kruskal-Wallis test was applied. Proportions were compared using the Fisher's exact test or the χ2 test, when appropriate. P < 0.05 was considered statistically significant. All data were analyzed by the SPSS software for Windows, Version 16.0 (SPSS Inc., Chicago, IL, USA).

## Abbreviations

PPOS: progestin-primed ovarian stimulation
COH: controlled ovarian hyperstimulation
FET: frozen-thawed embryo transfer
GnRH: gonadotropin-releasing hormone
hCG: human chorionic gonadotrophin
hMG: human menopausal gonadotropin
IVF: *in vitro* fertilization
ICSI: intracytoplasmic sperm injection
Gn: gonadotropin
LH: luteinizing hormone
FSH: follicle stimulation hormone
MC: menstruation cycle day
MPA: medroxy-progesterone acetate
P: progesterone
E_2_: estrogen
BMI: body mass index.

## References

[1] Leao Rde B, Esteves SC (2014). Gonadotropin therapy in assisted reproduction: an evolutionary perspective from biologics to biotech. Clinics (Sao Paulo).

[2] Ezcurra D, Humaidan P (2014). A review of luteinising hormone and human chorionic gonadotropin when used in assisted reproductive technology. Reprod Biol Endocrinol.

[3] Choi J, Smitz J (2014). Luteinizing hormone and human chorionic gonadotropin: origins of difference. Mol Cell Endocrinol.

[4] Riccetti L, Yvinec R, Klett D, Gallay N, Combarnous Y, Reiter E, Simoni M, Casarini L, Ayoub MA (2017). Human luteinizing hormone and chorionic gonadotropin display biased agonism at the LH and LH/CG receptors. Sci Rep.

[5] Cole LA (2012). hCG, the wonder of today's science. Reprod Biol Endocrinol.

[6] Casarini L, Lispi M, Longobardi S, Milosa F, La Marca A, Tagliasacchi D, Pignatti E, Simoni M LH and hCG action on the same receptor results in quantitatively and qualitatively different intracellular signalling. PloS One.

[7] van Wely M, Kwan I, Burt AL, Thomas J, Vail A, Van der Veen F, Al-Inany HG (2011). Recombinant versus urinary gonadotrophin for ovarian stimulation in assisted reproductive technology cycles. Cochrane Database Syst Rev.

[8] Martins WP, Vieira AD, Figueiredo JB, Nastri CO (2013). FSH replaced by low-dose hCG in the late follicular phase versus continued FSH for assisted reproductive techniques. The Cochrane Database of Systematic Reviews.

[9] Awwad JT, Farra C, Mitri F, Abdallah MA, Jaoudeh MA, Ghazeeri G (2013). Split daily recombinant human LH dose in hypogonadotrophic hypogonadism: a nonrandomized controlled pilot study. Reprod Biomed Online.

[10] Ashrafi M, Kiani K, Ghasemi A, Rastegar F, Nabavi M (2011). The effect of low dose human chorionic gonadotropin on follicular response and oocyte maturation in PCOS patients undergoing IVF cycles: a randomized clinical trial of efficacy and safety. Arch Gynecol Obstet.

[11] Madani T, Mohammadi Yeganeh L, Khodabakhshi S, Akhoond MR, Hasani F (2012). Efficacy of low dose hCG on oocyte maturity for ovarian stimulation in poor responder women undergoing intracytoplasmic sperm injection cycle: a randomized controlled trial. J Assist Reprod Genet.

[12] Massin N (2017). New stimulation regimens: endogenous and exogenous progesterone use to block the LH surge during ovarian stimulation for IVF. Hum Reprod Update.

[13] Kuang Y, Chen Q, Fu Y, Wang Y, Hong Q, Lyu Q, Ai A, Shoham Z (2015). Medroxyprogesterone acetate is an effective oral alternative for preventing premature luteinizing hormone surges in women undergoing controlled ovarian hyperstimulation for in vitro fertilization. Fertil Steril.

[14] Wang Y, Chen Q, Wang N, Chen H, Lyu Q, Kuang Y (2016). Controlled ovarian stimulation using medroxyprogesterone acetate and hMG in patients with polycystic ovary syndrome treated for IVF: a double-blind randomized crossover clinical trial. Medicine (Baltimore).

[15] Zhu X, Zhang X, Fu Y (2015). Utrogestan as an effective oral alternative for preventing premature luteinizing hormone surges in women undergoing controlled ovarian hyperstimulation for in vitro fertilization. Medicine (Baltimore).

[16] Zhu X, Ye H, Fu Y (2016). The Utrogestan and hMG protocol in patients with polycystic ovarian syndrome undergoing controlled ovarian hyperstimulation during IVF/ICSI treatments. Medicine (Baltimore).

[17] Zhu X, Ye H, Fu YL (2017). Use of Utrogestan during controlled ovarian hyperstimulation in normally ovulating women undergoing in vitro fertilization or intracytoplasmic sperm injection treatments in combination with a “freeze all” strategy: a randomized controlled dose-finding study of 100 mg versus 200 mg. Fertil Steril.

[18] van Wely M, Kwan I, Burt AL, Thomas J, Vail A, Van der Veen F, Al-Inany HG (2011). Recombinant versus urinary gonadotrophin for ovarian stimulation in assisted reproductive technology cycles. The Cochrane database of systematic reviews.

[19] Al-Inany HG, Abou-Setta AM, Aboulghar MA, Mansour RT, Serour GI Efficacy and safety of human menopausal gonadotrophins versus recombinant FSH: a meta-analysis. Reprod Biomed Online.2008;.

[20] Al-Inany H, van Gelder P (2010). Success of frozen embryo transfer: Does the type of gonadotropin influence the outcome?. Int J Womens Health.

[21] Lehert P, Schertz JC, Ezcurra D (2010). Recombinant human follicle-stimulating hormone produces more oocytes with a lower total dose per cycle in assisted reproductive technologies compared with highly purified human menopausal gonadotrophin: a meta-analysis. Reprod Biol Endocrinol.

[22] Brannian J, Eyster K, Mueller BA, Bietz MG, Hansen K (2010). Differential gene expression in human granulosa cells from recombinant FSH versus human menopausal gonadotropin ovarian stimulation protocols. Reprod Biol Endocrinol.

[23] Richter TA, Robinson JE, Evans NP (2001). Progesterone treatment that either blocks or augments the estradiol-induced gonadotropin-releasing hormone surge is associated with different patterns of hypothalamic neural activation. Neuroendocrinology.

[24] Goodman RL, Bittman EL, Foster DL, Karsch FJ (1981). The endocrine basis of the synergistic suppression of luteinizing hormone by estradiol and progesterone. Endocrinology.

[25] Ye H, Huang G, Pei L, Zeng P, Luo X (2012). Outcome of in vitro fertilization following stimulation with highly purified hMG or recombinant FSH in downregulated women of advanced reproductive age: a prospective, randomized and controlled trial. Gynecol Endocrinol.

[26] Beretsos P, Partsinevelos GA, Arabatzi E, Drakakis P, Mavrogianni D, Anagnostou E, Stefanidis K, Antsaklis A, Loutradis D (2009). “hCG priming” effect in controlled ovarian stimulation through a long protocol. Reprod Biol Endocrinol.

[27] Wang TH, Chang CL, Wu HM, Chiu YM, Chen CK, Wang HS (2006). Insulin-like growth factor-II (IGF-II), IGF-binding protein-3 (IGFBP-3), and IGFBP-4 in follicular fluid are associated with oocyte maturation and embryo development. Fertil Steril.

[28] Cole LA (2012). hCG, five independent molecules. Clin Chim Acta.

[29] Cole LA, DuToit S, Higgins TN (2011). Total hCG tests. Clin Chim Acta.

[30] Christensen H, Thyssen HH, Schebye O, Berget A (1990). Three highly sensitive “bedside” serum and urine tests for pregnancy compared. Clinical Chem.

[31] Elkind-Hirsch KE, Bello S, Esparcia L, Phillips K, Sheiko A, McNichol M (2001). Serum human chorionic gonadotropin levels are correlated with body mass index rather than route of administration in women undergoing in vitro fertilization--embryo transfer using human menopausal gonadotropin and intracytoplasmic sperm injection. Fertil Steril.

[32] Arce JC, Smitz J (2013). Live-birth rates after HP-hMG stimulation in the long GnRH agonist protocol: association with mid-follicular hCG and progesterone concentrations, but not with LH concentrations. Gynecol Endocrinol.

[33] Chan CC, Ng EH, Chan MM, Tang OS, Lau EY, Yeung WS, Ho PC (2003). Bioavailability of hCG after intramuscular or subcutaneous injection in obese and non-obese women. Hum Reprod.

[34] Alfthan H, Haglund C, Dabek J, Stenman UH Concentrations of human choriogonadotropin, its beta-subunit, and the core fragment of the beta-subunit in serum and urine of men and nonpregnant women. Clin Chem.1992;.

